# Hormone receptor-positive adenoid cystic carcinoma of the breast with cross-modality imaging discrepancy: a case report and literature review

**DOI:** 10.3389/fmed.2026.1966105

**Published:** 2026-10-13

**Authors:** Yunkuan Song, Yuezu He, Lu Peng, Fengjiang Qu, Sijie Li

**Affiliations:** 1 Department of Breast Surgery, General Surgery Center, The First Hospital of Jilin University, Changchun, China; 2 Department of Emergency Surgery, The First Hospital of Jilin University, Changchun, China

**Keywords:** adenoid cystic carcinoma, breast cancer, core needle biopsy, endocrine therapy, hormone receptor expression, immunohistochemistry

## Abstract

Primary adenoid cystic carcinoma of the breast (ACCB) is a rare salivary gland–type malignancy characterized by biphasic luminal epithelial and myoepithelial/basal differentiation. Although most ACCBs exhibit a triple-negative phenotype, hormone receptor–positive tumors occur, and evidence guiding endocrine therapy is limited. A 42-year-old woman presented with a gradually enlarging right breast mass. Ultrasonography and MRI were suspicious for malignancy (BI-RADS 4B and BI-RADS MRI category 5), whereas diagnostic mammography showed focal increased glandular density (BI-RADS 0) and benign punctate calcifications (BI-RADS 2). Core needle biopsy showed cribriform architecture, and immunohistochemistry demonstrated expression of CK7, CD117, and p63, supporting a diagnosis of ACCB. The biopsy showed estrogen receptor expression in 10%–20% of tumor cells, progesterone receptor expression in approximately 5%, and a HER2 score of 0. The patient underwent right mastectomy and sentinel lymph node biopsy. The resection specimen confirmed a 3.0 cm ACCB with predominantly cribriform architecture and additional solid growth, with ER and PR each expressed in approximately 30% of tumor cells, a HER2 IHC score of 0, and a negative sentinel lymph node [pT2 pN0(sn)]. She completed 5 years of adjuvant tamoxifen therapy. At the last follow-up, approximately 73 months after surgery, no local, regional, or distant recurrence was detected. This case highlights the value of integrating histomorphology and immunohistochemical findings when imaging findings differ across modalities and illustrates the immunophenotypic heterogeneity of ACCB. The recurrence-free outcome provides additional follow-up information for hormone receptor–positive ACCB but cannot establish benefit from endocrine therapy; individualized treatment and long-term surveillance remain warranted.

## 1 Introduction

Primary adenoid cystic carcinoma of the breast (ACCB) is a rare salivary gland-type malignancy included among rare and salivary gland-type tumours in the sixth edition of the WHO Classification of Breast Tumours (1). Although most ACCBs exhibit a triple-negative phenotype (2–7), hormone receptor expression has been reported in a subset of cases, for whom the clinical benefit of endocrine therapy remains uncertain (2, 5, 6, 8).

We report a case of primary ACCB in a 42-year-old woman. Core needle biopsy combined with immunohistochemistry supported the diagnosis preoperatively, and examination of the surgical specimen confirmed ACCB with predominantly cribriform architecture and a pathological stage of pT2 pN0(sn). Both specimens showed an HR-positive/HER2-negative immunophenotype, differing from the predominantly triple-negative phenotype reported in the literature. The patient completed 5 years of adjuvant tamoxifen therapy. At 73 months after surgery, there was no evidence of recurrence. This case illustrates the immunophenotypic heterogeneity of ACCB and provides additional clinical documentation of the diagnosis, endocrine treatment, and long-term follow-up of HR-positive disease. A focused PubMed search combined “adenoid cystic carcinoma” and “breast” with terms related to hormone-receptor expression, endocrine therapy, and multimodality imaging. Search terms included “estrogen receptor,” “progesterone receptor,” “tamoxifen,” “exemestane,” “mammography,” “ultrasound,” and “magnetic resonance imaging.” English-language reports published through September 2026 that provided relevant receptor, treatment, follow-up, or multimodality imaging data on ACCB were included. Reference lists of relevant articles were also screened to identify additional eligible reports. This case report was prepared in accordance with the CARE guidelines.

## 2 Case description

A 42-year-old woman presented to our hospital on April 9, 2020, with a right breast mass that had been incidentally detected 1 year earlier and had progressively increased in size. The patient had no personal or family history of malignancy. Physical examination revealed a firm, irregular, poorly circumscribed, and poorly mobile mass measuring approximately 3.0 × 2.0 cm in the upper inner quadrant of the right breast. No palpable enlarged lymph nodes were identified in the axillary or supraclavicular regions.

Ultrasonography showed a 31.6 × 10.9 mm hypoechoic mass in the upper inner quadrant of the right breast. The lesion had an irregular shape, indistinct margins, and internal vascularity and was classified as BI-RADS category 4B (Figure 1). No suspicious lymph nodes were identified in the right axilla. Diagnostic mammography showed heterogeneously dense breasts (breast-density category C). Focal increased glandular density was observed in the medial right breast and spatially corresponded to the palpable and ultrasonographic lesion. This finding was classified as BI-RADS category 0. Punctate calcifications in the same region were considered benign and were classified as BI-RADS category 2 (Figures 2A,B). Breast magnetic resonance imaging (MRI) showed a 1.6 × 1.9 cm nodular lesion in the upper inner quadrant. The lesion showed lobulated and spiculated margins, marked contrast enhancement, and rapid initial enhancement followed by a plateau (Figures 2C,D). It was classified as BI-RADS MRI category 5. All imaging examinations were completed on different dates before biopsy.

**Figure 1:**
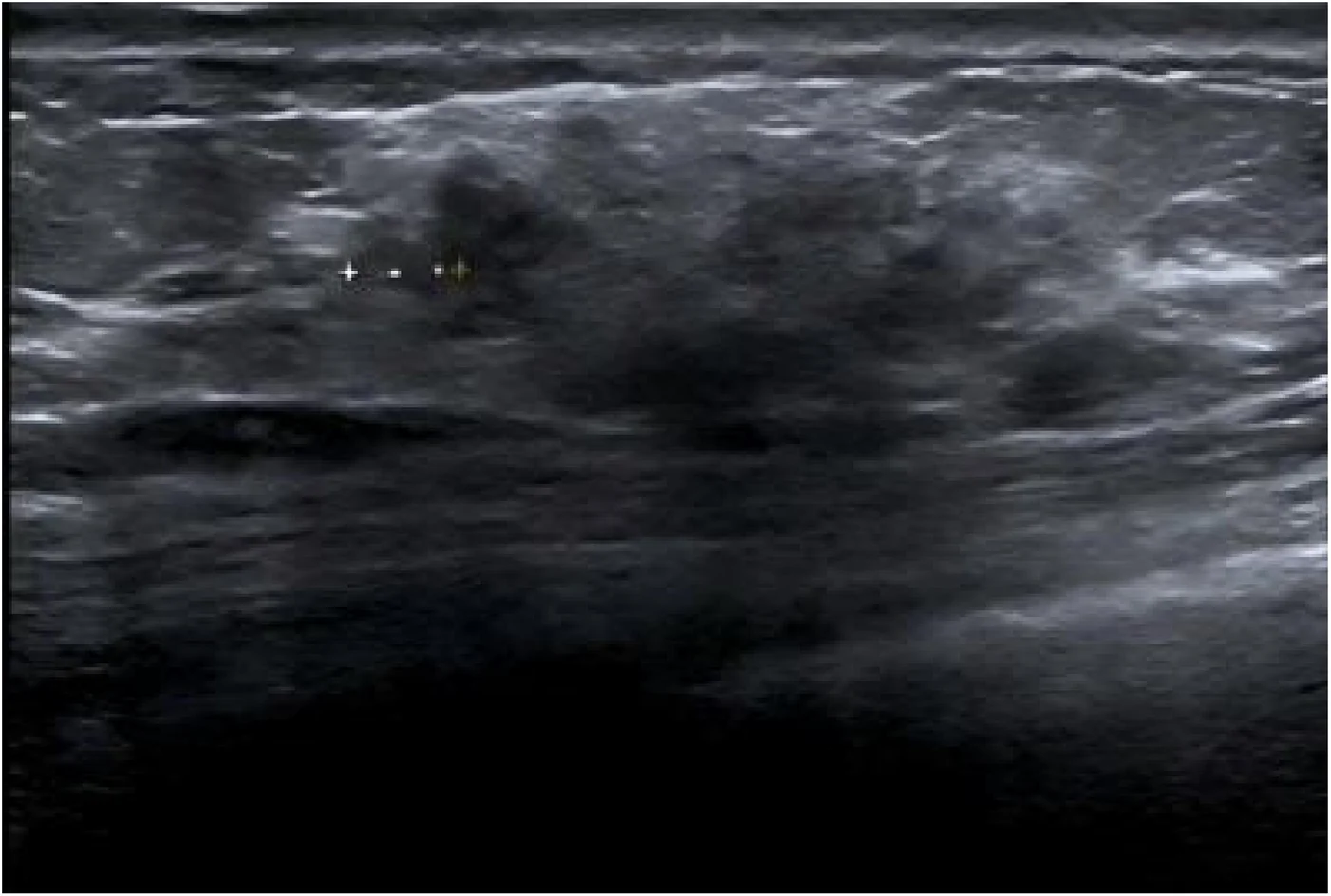
Grayscale ultrasonographic appearance of the right breast lesion. Ultrasonography showed a 31.6 × 10.9 mm irregular hypoechoic mass with indistinct margins in the upper inner quadrant. The lesion was classified as BI-RADS category 4B.

**Figure 2:**
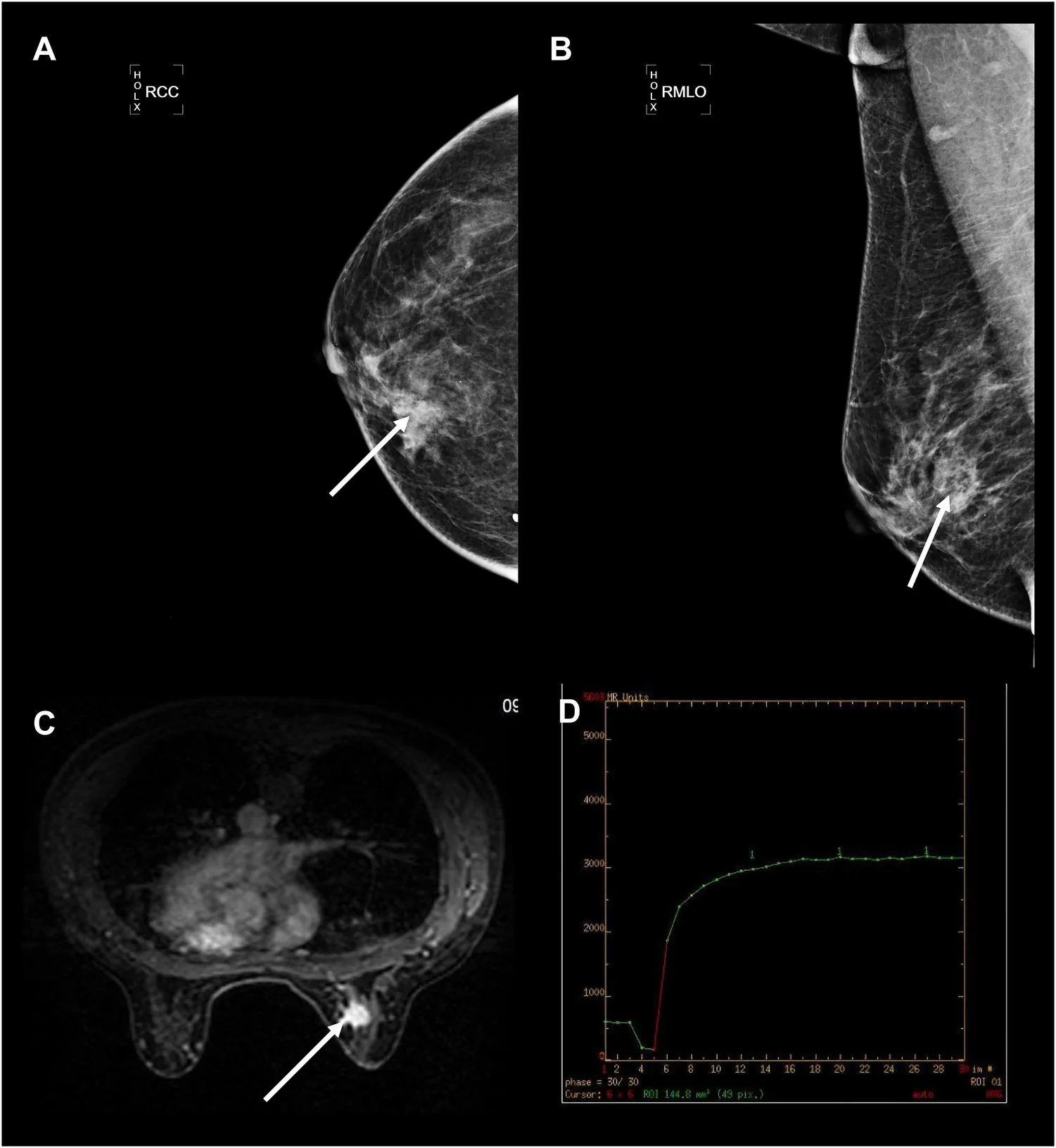
Mammographic and MRI findings of the right breast lesion. **(A)** Right craniocaudal (RCC) view. **(B)** Right mediolateral oblique (RMLO) view. The breast was heterogeneously dense. Focal increased glandular density was observed in the medial right breast. Punctate calcifications in the same region were considered benign. Arrows indicate the area corresponding to the palpable and ultrasonographic lesion. **(C)** Postcontrast MRI showing a markedly enhancing nodular lesion with lobulated and spiculated margins in the upper inner quadrant of the right breast (arrow). **(D)** Time–signal intensity curve showing rapid initial enhancement followed by a plateau.

Based on the clinical and imaging findings, a malignant breast tumor was considered highly likely. Core needle biopsy of the right breast mass was performed on April 20, 2020. Histologically, the core biopsy showed cribriform/microcystic and solid growth, myxoid material within pseudolumina, and focal basaloid cytomorphology (Figure 3C). The histological and immunohistochemical findings supported a diagnosis of ACCB. Immunohistochemistry showed nuclear ER staining in 10%–20% of tumor cells, PR expression in approximately 5% of tumor cells, a HER2 IHC score of 0. CK7 and CD117 were positive (Figure 3D). p63 showed nuclear staining within the tumor nests (Figure 3E), and CK5/6 showed scattered positivity. Post-biopsy radiologic-pathologic correlation indicated that the pathological findings were concordant with the targeted imaging abnormality. Subsequent systemic evaluation, including chest and abdominal computed tomography and whole-body bone scintigraphy, revealed no evidence of an extramammary primary tumor or distant metastasis. The tumor was clinically staged as cT2 cN0 cM0, and no contraindication to surgery was identified. After discussion of the available surgical options, the patient declined breast-conserving surgery and opted for mastectomy.

**Figure 3:**
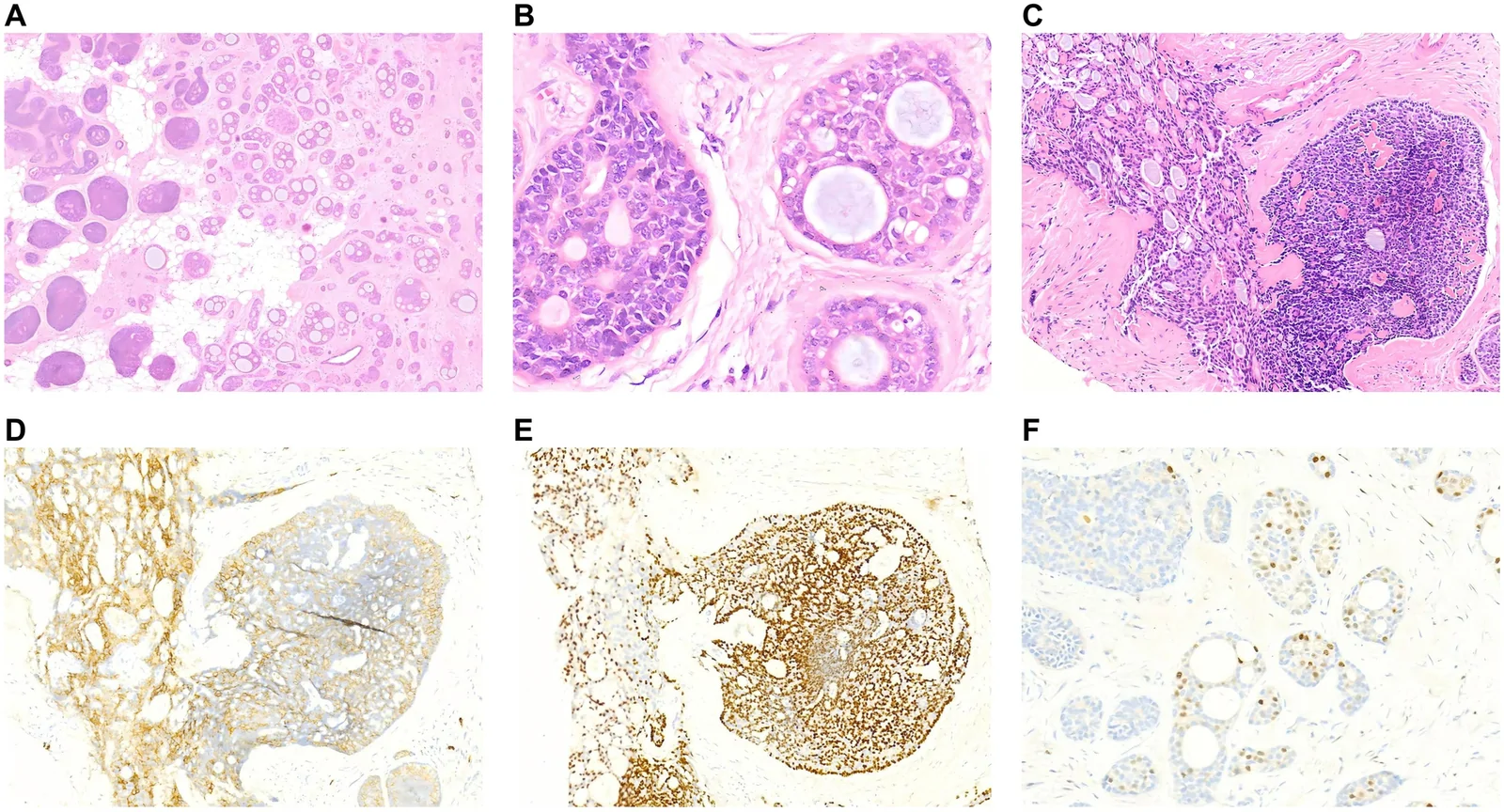
Histopathological and immunohistochemical features of adenoid cystic carcinoma of the breast. **(A)** Low-power view of the resection specimen showing solid and cribriform growth patterns and the interface between the tumor and adjacent adipose tissue (H&E, ×40). **(B)** High-power view showing true lumina lined by eosinophilic epithelial cells and pseudolumina containing basement membrane-like material surrounded by basaloid/myoepithelial cells with scant cytoplasm and hyperchromatic nuclei (H&E, ×400). **(C)** Core-biopsy specimen showing solid growth and focal basaloid cytomorphology characterized by scant cytoplasm and hyperchromatic nuclei (H&E, ×200). **(D)** CD117 immunostaining showing positivity in tumor cells in the core-biopsy specimen (×200). **(E)** p63 immunostaining showing nuclear positivity within tumor nests in the core-biopsy specimen (×200). **(F)** ER immunostaining showing nuclear positivity in approximately 30% of tumor cells within morphologically identified ACCB tumor nests in the resection specimen (×200).

On April 28, 2020, the patient underwent right mastectomy and sentinel lymph node biopsy under general anesthesia. One sentinel lymph node was submitted for intraoperative examination and was negative for metastasis. The procedure was completed without complications. Final histopathological examination revealed a 3.0 × 2.0 × 1.5 cm ACCB in the upper inner quadrant of the right breast, with predominantly cribriform architecture and additional solid growth (Figure 3A). At higher magnification, true lumina lined by epithelial cells with eosinophilic cytoplasm and pseudolumina containing basement membrane-like material were identified. The pseudolumina were surrounded by basaloid/myoepithelial cells with scant clear cytoplasm and hyperchromatic nuclei. The intervening solid areas contained luminal epithelial and basaloid/myoepithelial cell populations (Figure 3B). The tumor was assigned a Modified Bloom–Richardson–Nottingham grade of 2, although the individual component scores were not recorded. No definite lymphovascular invasion was identified. Tumor tissue focally surrounded a nerve, without definite perineural invasion. Gross examination showed that the tumor was 0.5 cm from the superficial fascia. No tumor involvement of the nipple or skin was identified, and no carcinoma was found in the remaining breast quadrants. Final permanent-section examination confirmed no metastasis in one sentinel lymph node (0/1). In the resection specimen, p63 showed nuclear staining within the tumor nests. ER showed nuclear staining in approximately 30% of tumor cells within morphologically identified ACCB tumor nests (Figure 3F), and PR was expressed in approximately 30% of tumor cells; the HER2 IHC score was 0. CD117 and CK7 were positive. Calponin, CK5/6, S-100, and smooth muscle actin (SMA) were positive in a subset of tumor cells.

Based on these findings, the final diagnosis was primary ACCB of the upper inner quadrant of the right breast, with an HR-positive/HER2-negative immunophenotype and a pathological stage of pT2 pN0(sn) according to the eighth edition of the AJCC staging system. The patient was premenopausal and received tamoxifen 20 mg once daily for 5 years without interruption. Chemotherapy and radiotherapy were not administered. Follow-up was performed every 6 months, with breast ultrasonography and contralateral mammography at 6- to 12-month intervals. Interval surveillance included chest and abdominal imaging and bone scintigraphy. At the last follow-up on May 24, 2026, approximately 73 months after surgery, there was no evidence of local, regional, or distant recurrence. Continued long-term follow-up was planned. The clinical course, diagnosis, treatment, and follow-up are summarized in Figure 4.

**Figure 4:**
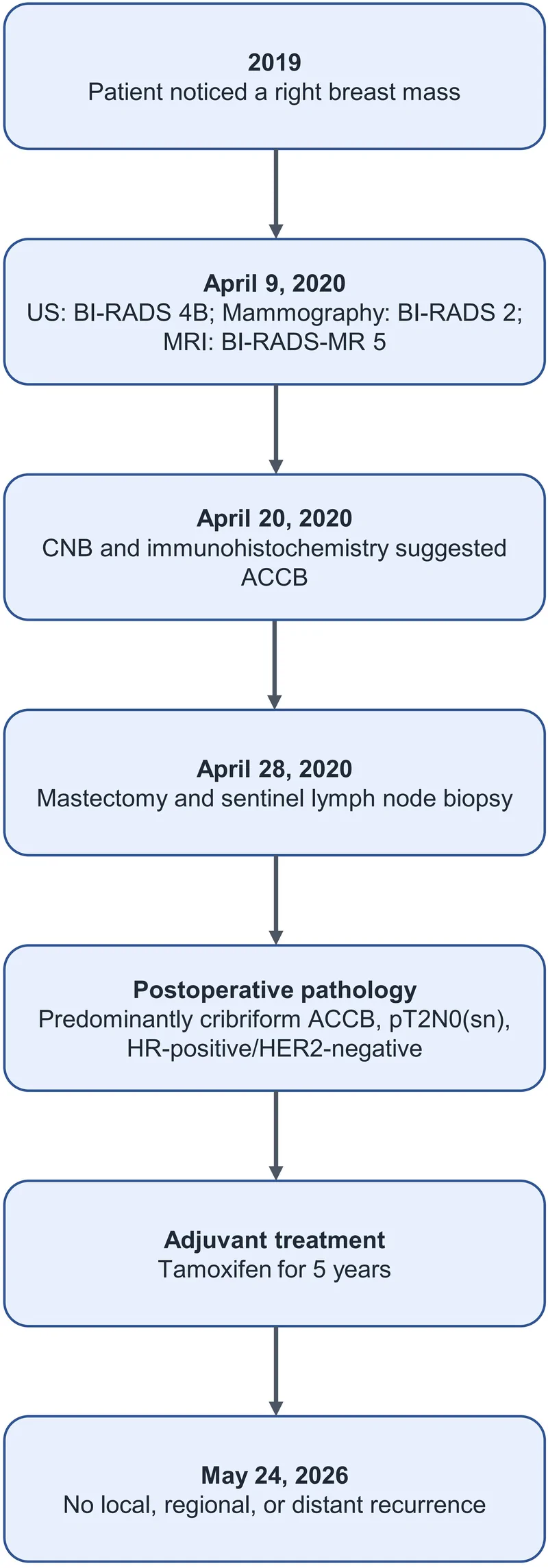
Timeline of the clinical course, diagnosis, treatment, and follow-up. ACCB, adenoid cystic carcinoma of the breast; CNB, core needle biopsy; HR, hormone receptor; MRI, magnetic resonance imaging; US, ultrasonography.

## 3 Patient perspective

At the latest follow-up, the patient expressed satisfaction with the treatment and current outcome and stated that she would continue regular follow-up.

## 4 Discussion

ACCB accounts for less than 0.1% of breast malignancies (2, 3, 6). A U.S. population-based study reported a median diagnostic age of 62 years and localized disease in approximately 95% of patients, with uncommon regional or distant involvement (9). In a Chinese single-institution series, ACCB comprised 14 of 23,205 breast cancers, and the median age was 60.5 years (5). Our patient was younger at diagnosis (42 years), and her tumor arose in the upper inner quadrant rather than the central or periareolar region described in some reports (2, 6). The clinical presentation of ACCB remains nonspecific.

The imaging features of ACCB are also nonspecific: lesions may appear malignant or resemble benign masses (10–12). In this case, ultrasonography and MRI were suspicious (BI-RADS 4B and BI-RADS MRI category 5), whereas mammography showed corresponding focal increased glandular density (BI-RADS 0) and benign punctate calcifications (BI-RADS 2). The lesion was therefore under-characterized, not occult, on mammography. Maximum diameters were 31.6 mm on ultrasonography, 19 mm on MRI, and 30 mm on pathological examination. The ultrasonographic measurement supported cT2 staging and approximated the pathological size. Cross-modality variation may reflect differences in imaging planes, lesion conspicuity, and measurement methods. Similar discrepancies have been reported (13, 14).

Core biopsy may not fully represent the cribriform architecture and biphasic components of ACCB (2, 6). In the 14-patient series by Zhang et al., all six fine-needle aspiration specimens raised suspicion for ACCB, whereas one of four core biopsies was reported only as invasive carcinoma when immunohistochemistry was not performed (5). In our case, supportive morphology and immunohistochemistry permitted a preoperative diagnosis. Typical ACCB contains luminal epithelial and myoepithelial/basaloid cells forming true lumina and basement membrane-containing pseudolumina. Luminal cells commonly express CK7 and CD117 (KIT), whereas the myoepithelial/basaloid population may express p63, CK5/6, S-100, SMA, and calponin (3, 6, 15, 16). CK7 and CD117 positivity, nuclear p63 staining within tumor nests, and scattered CK5/6 positivity supported the biopsy diagnosis. S-100, SMA, and calponin positivity in a subset of cells provided additional support in the resection specimen.

The differential diagnosis of ACCB includes invasive cribriform carcinoma, tubular carcinoma, cribriform ductal carcinoma *in situ* (DCIS), cribriform-pattern invasive carcinoma of no special type (NST), collagenous spherulosis, and adenomyoepithelioma (6, 15–17). ACCB shows an internal biphasic cell population and basement membrane-like pseudolumina (2, 6, 15). Invasive cribriform carcinoma shows a single luminal cell population. Tubular carcinoma is composed of angulated tubules lined by a single epithelial layer (6, 16). Cribriform-pattern NST lacks the characteristic internal biphasic pattern of ACCB. Cribriform DCIS retains a peripheral myoepithelial layer. Collagenous spherulosis is a benign mimic with collagenous spherules (6, 17). Adenomyoepithelioma and adenomyoepithelial adenosis are other biphasic lesions but differ from ACCB in their overall architecture. Solid-basaloid ACCB and high-grade transformation may show greater atypia, mitotic activity, or necrosis (18, 19). Metastatic adenoid cystic carcinoma requires exclusion of an extramammary primary tumor (2, 15). Diagnosis should integrate morphology, marker distribution, and the overall immunohistochemical profile rather than rely on CD117 or p63 alone (6, 16). The resection specimen showed predominantly cribriform architecture with additional solid growth. No definite lymphovascular invasion was identified. Tumor tissue focally surrounded a nerve, without definite perineural invasion. The proportions of the solid and basaloid components were not recorded, limiting more detailed histological risk stratification. *MYB::NFIB* fusion is a recurrent molecular alteration in ACCB (20, 21). In a study of four breast ACCBs negative for the *MYB::NFIB* fusion, *MYBL*1 rearrangements were identified in two tumors, whereas high-level *MYB* amplification was detected in one (22). Molecular testing is not required in morphologically typical cases but may provide ancillary diagnostic support when the histological findings are atypical or the differential diagnosis remains unresolved (15, 20–22).

Although ACCB most commonly exhibits a triple-negative phenotype (2–6), the present case was HR-positive/HER2-negative. In a Chinese single-institution series, 6 of 14 patients had HR-positive tumors (5). In a SEER-based study of 661 surgically treated patients with ACCB, 20.7% of tumors were ER-positive, and ER status was not significantly associated with either overall survival or breast cancer-specific survival in univariable analyses (4). The former study had a small sample size, whereas the latter lacked centralized pathological review and detailed information on the percentage of receptor-positive tumor cells. These findings indicate that hormone receptor expression occurs in a subset of ACCBs but do not define its precise prevalence or prognostic significance. In the present case, the core biopsy showed ER expression in approximately 10%–20% of tumor cells and PR expression in approximately 5%, whereas the surgical specimen showed ER and PR expression in approximately 30% of tumor cells. HER2 was scored as 0 in both specimens. Core-to-resection receptor differences may reflect sampling variation, intratumoral heterogeneity, or technical variation rather than biological evolution. Both ER and PR results met ASCO/CAP criteria for positivity (23); together with a HER2 score of 0, these findings supported classification as HR-positive/HER2-negative.

Surgery is the mainstay of treatment, but breast-conserving surgery and mastectomy have not been compared prospectively (2, 24, 25). In a SEER study of 583 stage I–II cases, including 386 treated with breast-conserving surgery, 10-year overall survival was comparable between procedures (25). Higher disease-specific survival after breast conservation was confounded by differences in stage, grade, and radiotherapy and does not establish superiority. Retrospective series support breast-conserving surgery with negative margins, usually followed by radiotherapy, for localized disease (24, 26, 27). Selection should consider tumor extent, tumor-to-breast size ratio, margin feasibility, cosmesis, and patient preference. Our patient chose mastectomy after both options were discussed. Grossly, the tumor was 0.5 cm from the superficial fascia. No nipple or skin involvement or carcinoma in other quadrants was found, and no local recurrence occurred during follow-up.

Regional lymph node metastasis is uncommon in ACCB, but no standardized approach to axillary surgery has been established. In the California Cancer Registry, 12 of 244 patients (5%) had regional disease, although this registry category was not equivalent to pathologically confirmed nodal metastasis (28). In the National Cancer Data Base series of 933 patients, 75.9% underwent axillary evaluation and 5.1% had node-positive disease (29). A centrally reviewed series of 20 pure ACCBs reported no nodal metastases among the 15 patients who underwent sentinel lymph node biopsy and no regional nodal recurrences during follow-up (30). These findings do not support routine axillary lymph node dissection in patients with a clinically negative axilla. Sentinel lymph node biopsy remains a less invasive staging option; however, whether it can be safely omitted in selected patients with clinically node-negative ACCB remains uncertain. In the present case, neither physical examination nor preoperative imaging revealed suspicious axillary lymph nodes. One sentinel lymph node was negative for metastasis, and no further axillary dissection was performed. This approach was consistent with the available retrospective evidence, although the evidence remains insufficient to determine whether sentinel lymph node biopsy could have been safely omitted.

Prospective evidence on adjuvant treatment for ACCB is lacking, and clinical decisions rely mainly on retrospective data and treatment principles established for more common breast cancers. In the Rare Cancer Network cohort, radiotherapy after breast-conserving surgery was associated with a higher 5-year locoregional control rate than surgery without radiotherapy (95% vs. 83%; *p* = 0.03) (24). By contrast, SEER-based studies evaluated survival rather than recurrence and reported associations between postoperative radiotherapy and improved overall and/or cause-specific survival (4, 31, 32). A recent multicenter study found no significant association between postoperative radiotherapy and locoregional recurrence-free survival; however, only four patients treated with breast-conserving surgery omitted radiotherapy, limiting the strength of this comparison (27). Collectively, these retrospective findings do not establish a causal benefit of radiotherapy. Evidence regarding postmastectomy radiotherapy remains particularly limited. Postmastectomy radiotherapy was not administered in the present patient with pT2 pN0(sn) disease. The absence of recurrence in this single case should not be interpreted as evidence that radiotherapy can be routinely omitted in similar patients. A SEER-based analysis of 661 surgically treated patients with ACCB found no significant differences in overall or breast cancer-specific survival between patients who received chemotherapy and those who did not after 1:1 propensity score matching, with 82 patients in each matched group (4). The SEER dataset lacked information on chemotherapy regimens, recurrence outcomes, and centralized pathological review, and residual treatment-selection bias could not be excluded. These findings are consistent with the decision not to administer chemotherapy in the present patient with pT2 pN0(sn) ACCB but do not establish a lack of benefit in higher-risk settings, such as lymph node-positive disease, solid-basaloid ACCB, high-grade transformation, or advanced-stage disease.

No prospective trials have specifically evaluated endocrine therapy for HR-positive ACCB. Previous reports have described ER-low tumors treated with tamoxifen (33, 34). Yan et al. reported recurrence-free follow-up to 160 months (33). In Zhang et al., five of six HR-positive patients received endocrine therapy, with 32–95 months of follow-up (5). Chen et al. reported hormone therapy with tamoxifen or exemestane in two HR-positive patients (35). These reports establish precedent but do not demonstrate treatment efficacy. Our patient completed five years of tamoxifen and remained recurrence-free at 73 months. This outcome cannot be attributed solely to endocrine therapy. ACCB generally has a favorable prognosis. Ghabach et al. reported 5-, 10-, and 15-year relative survival rates of 98.1%, 94.9%, and 91.4%, respectively (9). However, high relative survival does not imply an absence of recurrence risk, and the SEER database did not capture recurrence. Reports of local recurrence or distant metastasis many years after initial treatment underscore the need for long-term follow-up. The lung is the most commonly reported site of distant metastasis, although bone and liver involvement can also occur (2, 6). Large morphologically reviewed studies have associated basaloid morphology, higher histological grade, lymphovascular or perineural invasion, necrosis, positive margins, lymph node involvement, and greater solid growth with recurrence or shorter recurrence-free survival. However, their definitions and independent prognostic effects have varied across studies (18, 36, 37). In the present case, the tumor showed predominantly cribriform architecture with additional solid growth and was node-negative. No definite lymphovascular invasion was identified. Tumor tissue focally surrounded a nerve, without definite perineural invasion. No local, regional, or distant recurrence was observed during approximately 73 months of follow-up. Continued long-term surveillance is warranted because late recurrence can occur.

This report has several limitations. A single case cannot establish tamoxifen efficacy, and the absence of recurrence cannot be attributed solely to endocrine therapy. *MYB/MYBL*1 testing was not performed, and some histopathological variables could not be assessed retrospectively because complete slides and records were unavailable. Nevertheless, the case documents a preoperative core-biopsy diagnosis and illustrates cross-modality imaging differences and an uncommon HR-positive/HER2-negative immunophenotype. Treatment decisions should integrate imaging, histology, immunophenotype, pathological risk factors, and patient preference.

## 5 Conclusion

The diagnosis of ACCB rests on characteristic histological features supported by immunohistochemistry. Although the findings differed across imaging modalities in this case, core needle biopsy combined with immunohistochemistry strongly supported the diagnosis of ACCB preoperatively, underscoring the value of integrated radiologic-pathologic assessment. The ER-positive, PR-positive, and HER2-negative immunophenotype in this case illustrates heterogeneity in receptor expression among ACCBs. The patient completed 5 years of tamoxifen therapy and remained recurrence-free during follow-up, but a single case cannot determine the benefit of endocrine therapy. Treatment decisions should reflect histological features, stage, margin status, and other relevant risk factors. Despite the generally favorable prognosis reported for ACCB, long-term surveillance is necessary because late recurrence can occur.

## Data Availability

The raw data supporting the conclusions of this article will be made available by the authors, without undue reservation.
